# Local Genetic Signatures of a Global Weed: Population Structure and Ecological Drivers of *Capsella bursa‐pastoris* Diversity in the Korean Peninsula

**DOI:** 10.1002/ece3.73980

**Published:** 2026-08-18

**Authors:** Byungwook Choi, Eunsuk Kim, Peter Tiffin, Sang‐Tae Kim

**Affiliations:** ^1^ Department of Plant and Microbial Biology University of Minnesota St. Paul Minnesota USA; ^2^ Department of Environmental and Energy Engineering Gwangju Institute of Science and Technology Gwangju Republic of Korea; ^3^ Department of Medical and Biological Sciences The Catholic University of Korea Bucheon Republic of Korea

**Keywords:** admixture, gene flow, population differentiation, RAD‐seq

## Abstract

Genetic variation in widespread plant species is shaped by adaptation to local environments and gene flow among subpopulations. *Capsella bursa‐pastoris* is a globally distributed weedy species and a model for studying polyploid genome evolution. The demographic history of European populations has been well characterized, yet East Asian populations of *C. bursa‐pastoris* are poorly explored. Here, we use RADseq data to investigate the genetic diversity and population structure of 187 *C. bursa‐pastoris* accessions across the Korean Peninsula, a region of mountainous terrain and extensive ecological heterogeneity. In contrast to European populations, which show high rates of selfing, we found that Korean populations exhibited signs of outcrossing, potentially resulting from high population density influenced by human activities embedded in Korean history and culture. Widespread genetic admixture contributed to the overall low genetic differentiation, as multiple introgression events into Korea may have occurred, followed by extensive gene flow among populations mediated by human activities. The genetic differentiation that was detected was largely correlated with geographic and climatic divergence. Taken together, our findings highlight the potential for range expansions to be accompanied by shifts in mating systems and suggest that Korean *C. bursa‐pastoris* populations provide an ecological model for studying the transition from selfing to outcrossing.

## Graphical abstract

*Capsella bursa‐pastoris* is a globally distributed weedy species widely used to study polyploid genome evolution, yet its East Asian populations remain poorly understood. Using RADseq from 187 accessions across the Korean Peninsula, we found evidence of outcrossing and extensive genetic admixture, likely driven by human‐mediated gene flow. These results suggest a shift in mating system during range expansion and highlight Korean populations as a model for studying transitions from selfing to outcrossing.

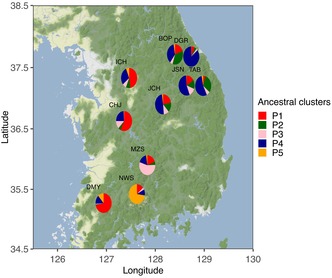

## 1 Introduction

According to Baker's law, species capable of self‐fertilization (selfing) are more likely to successfully colonize and establish in new environments following long‐distance dispersal (Baker 1955; Pannell 2016; Pannell et al. 2015). This success is facilitated by reproductive assurance, a reproductive advantage when mates and/or pollinators are scarce (Eckert et al. 2006; Grossenbacher et al. 2015; Jain 1976). Predominantly selfing species also tend to exhibit genetic differentiation at finer spatial scales than outcrossers, as they can adapt to local habitat conditions that vary even over relatively small geographic distances (Bomblies et al. 2010; Linhart and Grant 1996).

*Capsella bursa‐pastoris* (L.) Medik is one of the most extensively studied globally distributed weedy species, which is an allotetraploid species (2*n* = 4*x* = 32) that originated through hybridization between *C. orientalis* and *C. rubella*/ *C. grandiflora* and is considered to be predominantly selfing (Hintz et al. 2006; Hurka and Neuffer 1997; Douglas et al. 2015). The study of *C. bursa‐pastoris* has focused primarily on European populations, which have served as an important model for studies on polyploid genome evolution involving hybridization (Bachmann et al. 2021; Douglas et al. 2015; Kasianov et al. 2017; Kryvokhyzha et al. 2019; Penin et al. 2024), phenotypic plasticity (Choi et al. 2019; Cornille et al. 2022; Hurka and Neuffer 1997; Neuffer 2011), as well as the spatial and climatic factors contributing to genetic variation at a global scale (Han et al. 2015; Wesse et al. 2021), and characterizing the demographic history of weedy plants (Cornille et al. 2016; Penin et al. 2024).

Asian populations of *C. bursa‐pastoris* have been characterized far less extensively than European populations, despite being included in a few demographic studies at the global scale (i.e., Wesse et al. 2021). A major goal of this work was to expand the sampling range and conduct subsequent population genetic analyses at finer regional scales within East Asia, particularly in underrepresented Korean *C. bursa‐pastoris* populations, which are thought to have been introduced approximately 1000 years ago (Cornille et al. 2016). These populations provide an opportunity to enhance our understanding of the species' colonization history (Cornille et al. 2016) and how climatic factors shape population structure (Choi et al. 2019).

The ecological context of *C. bursa‐pastoris* (pronounced in Korean as Naeng‐i [nɛŋɡ:i]) in Korea is likely shaped by the geographical landscape, ecological factors, and human activities. Due to geopolitical isolation following the Korean war, the Korean Peninsula has experienced reduced terrestrial connectivity with continental Eurasia following geopolitical division, potentially limiting recent plant dispersal and introduction from outside. In addition, the Baekdu‐daegan mountain range, which covers approximately 70% of the Korean Peninsula (Choe et al. 2016), creates a complex ecological and elevational landscape that contributes to climatic heterogeneity (Cho et al. 2024) and serves as a dispersal barrier for both plants and animals. *Capsella bursa‐pastoris* also has been used in diverse Korean cuisines and traditional remedy materials for centuries. It is referenced in *Dong‐ui‐bo‐gam*, one of the oldest medical encyclopedia in East Asia (Song et al. 2016), where it was recognized both as a nutritious food source and as a material for traditional medicine (Ahmed et al. 2022; Cha et al. 2018; Hur et al. 2013; Jeong et al. 2023). *Capsella bursa‐pastoris* still remains in high demand in contemporary Korea, where it is frequently traded in wholesale and retail markets and typically harvested from wild fields, combined with widespread collection and distribution through local markets and human mediated transport (Pemberton and Lee 1996). Such human‐mediated activities including the collection and redistribution of wild plants have likely facilitated gene flow among populations and may be responsible for its widespread presence in diverse habitats including fallow fields and paddies.

We propose that the anthropogenic activities and highly heterogeneous environment found in the Korean Peninsula may have strongly shaped the evolution of Korean *C. bursa‐pastoris* . Here, we conducted population genomic analyses of Korean *C. bursa‐pastoris* using a RADseq approach to characterize: (i) the extent of genetic diversity within and among natural populations, (ii) the role of altitude and geographic distance in genetic structure and (iii) the ecological factors that most strongly contribute to genetic divergences among populations within the Korean Peninsula. Our analyses reveal that Korean populations exhibit distinct genetic and ecological patterns, including widespread outcrossing in a species previously considered predominantly selfing. These findings provide a more comprehensive understanding of the evolutionary dynamics of this globally distributed weedy species.

## 2 Materials and Methods

### 2.1 Seed Sources Collection

Ten natural populations of *C. bursa‐pastoris* , spanning the broad altitudinal range in the Korean Peninsula were chosen for sampling; three populations (DMY, CHJ, and ICH) are “LOW” of altitude lower than 100 m, three populations (JSN, JCH, and NWS) are “MID” of altitude between 300 and 400 m, and four populations (BOP, DGR, MZS, and TAB) are “HIGH” of altitude higher than 700 m across the Korean Peninsula (Table S1 and Figure S1). From May to June 2018, seeds were collected from at least 20 individuals from each population, with at least 3 m interval among each sampled individual.

### 2.2 Library Preparation and Sequencing

The seeds collected from each population were placed on soil in a growth chamber under 25°C/15°C with light on/off (16/8 h) for 3 weeks. Fully expanded, healthy rosette leaves were harvested for DNA extraction following Edwards et al. (1991). Preparation of RADseq libraries was carried out for 187 individuals (12–32 per population, average 18.7, Table S1) following the protocol in Rowan et al. (2017). The extracted DNA was digested with KpnI‐HF enzymes followed by ligation with T4 ligase. The 187‐plex pooled sample was purified using SPRI beads, and the pooled DNA was fragmented using a sonicator (Q800R2, Qsonica). DNA amplification was performed with Phusion HF Master Mix (#M0541, NEB). The amplicon size (mean = 544 bp) of the prepared library was quantified using DNA 1000 kit (#5057‐1504) for Agilent Bioanalyzer 2100. Finally, the library was sequenced using the Illumina Hiseq2500 50SE by Macrogen Inc. (Korea). Details regarding library preparation are described in Supporting Information.

### 2.3 Raw Data Filtering and Genotyping

Raw reads (51 bp length) were demultiplexed into each population based on the barcode reads (1.2 M mean read counts, Dataset S1), and then enzyme sites were trimmed using *process_radtags* from Stacks2 (Rochette et al. 2019). We genotyped the loci from the trimmed sequences using the Stacks2 pipeline, followed by polyRAD processes for polyploid species described by Salvado et al. (2022). Firstly, sequences were processed using *ustacks* with *m* = 3, *M* = *n* = 4, and *max_locus_stacks* = 5, where *m* represents the minimum number of identical reads required to form a putative allele, *M* represents the maximum number of mismatches allowed between alleles within a locus, *n* represents the number of mismatches allowed between loci during construction of the catalog, and *max_locus_stacks* represents the maximum number of stacks permitted at a single locus. *Cstacks* with *n* = 5, where *n* represents the number of mismatches allowed between sample loci when building the catalog, followed by matching individual loci to the catalog using sstacks were performed (Rochette et al. 2019). Secondly, we employed polyRAD (Clark et al. 2019), a R package to call genotypes from sequencing data in polyploids through adjusting *possiblePloidies*. In PolyRAD, we set the min.ind.with.minor.alleles = 168, min.ind.with.reads = 10, and possiblePloidy of list (2), indicating that the allopolyploid genome was modeled as disomically inherited, forming bivalents during meiosis (St. Onge et al. 2012), because *C. bursa–pastoris* has been recognized as an allotetraploid species (Hurka et al. 1989; Kasianov et al. 2017; Kryvokhyzha et al. 2019). The retained 4681 variants were filtered based on the distribution of the ratio of individual over expected heterozygosity (*H*_Ind_/*H*_E_). We used *H*_Ind_/*H*_E_ to filter individuals processed by depth for each sample which was calculated with rowMeans across all samples. Samples presenting *hhByInd* < 0.45 were removed, resulting in the exclusion of six samples with the lowest overall coverage (< 7.5%; see Dataset S1). After samples filtering, loci were filtered based on the distribution of *H*_Ind_/*H*_E_ values calculated using *ExpectedHindHe*() in polyRAD (reps = 100, overdispersion = 130). We retained loci with *H*_Ind_/*H*_E_ values ranging from 0.428 to 0.575 (mean = 0.502), removing the top 82.4% and bottom 12.6% of variants. This filtering resulted in a final dataset of 233 SNPs used for subsequent analysis.

### 2.4 Genetic Diversity and Population Structure Analysis

We calculated the observed and expected heterozygosity (*H*_O_ and *H*_E_), and inbreeding coefficients (*F*_IS_) for each population using *basic. stats()* in the *hierfstat* R package. We also calculated F statistics for differentiation between populations using *mat_gen_dist()* (dist = “FST”) by graph4lg R package (Savary et al. 2021). To understand the genetic structure among the samples, two scatter plots were constructed (i) principal component analysis (PCA) without considering any a priori population definition using *dudi.pca()* from *ade4* R package (Chessel et al. 2009), and (ii) discriminant analysis of principal components (DAPC), with synthetic discriminant axes that maximize among‐population while minimizing within‐population variation, was performed using *dapc()* from *adegenet* R package (Jombart et al. 2010). We also performed *snmf()* in *LEA* R package to estimate the most likely number of ancestral genetic clusters for values of *K* = 1 to 20 with the “geno” object created from the filtered vcf type of datamatrix by *vcf2geno()* in *LEA* package (Frichot and François 2015), and analyses were run with 200 replicates at each value of *K*. We determined *K* = 5 using *cross. entropy()*, which was selected where the cross‐entropy difference starts to plateau. Finally, the structuring of variation within and among populations was estimated by Analysis of Molecular Variance (AMOVA) using *poppr.amova()* in *poppr* package (Excoffier et al. 1992), which was performed with 9999 permutations.

### 2.5 Spatial and Environmental Drivers Contributing to Genetic Differentiation

To characterize how genetic differentiation between populations is driven by the geographical distances (IBD; Isolation by Distance) and environmental gradients (IBE; Isolation by Environment), we performed a Mantel test. Firstly, the genetic distances between populations were calculated as linearized *F*_ST_ using *mat_gen_dist()* (dist = “LinFST”) by *graph4lg* R package (Savary et al. 2021). For the geographic distances, we calculated the distances between population pairs from geographic coordinates (Table 1) using *distm()* (fun = distHarversine) in *geosphere* R package (Hijmans et al. 2017). We also set the relative spatial distances of elevation, latitude, and longitude between populations by normalization using the *distance()* function with “euclidean” in *ecodist* R package (Goslee and Urban 2007). For environmental variables for each population, we collected 19 climatic variables defined by the WorldClim database (Fick and Hijmans 2017; see Table S2), which were calculated with monthly‐records from AWS (Automatic Weather Station) operated by KMA (Korea Meteorological Administration) from Jan 1997 to Dec 2018. Since not all populations had AWS at the exact location that we harvested seeds, we used environmental data of two populations (BOP and MZS) from the closest locations of similar altitude near our sampling site. From the 19 climatic variables, we standardized the environmental variables using *scale()*, and then calculated the environmental distance matrices using *distance()* function with “gower” in *ecodist* R package. Mantel tests were carried out separately for the spatial distances and each of the 19 environmental distances using *mantel()* with 9999 permutations in *vegan* R package (Oksanen et al. 2013).

**TABLE 1:** Genetic diversity indices for 10 *Capsella bursa‐pastoris* populations, including observed heterozygosity (*H*_O_), expected heterozygosity (*H*_E_), and inbreeding coefficient (*F*_IS_), calculated using stacks2‐polyRAD pipeline. Altitudinal group classification (Groups) based on elevation is also shown.

Populations	Groups	*H*_O_	*H*_E_	*F*_IS_
BOP	HIGH	0.30	0.24	−0.16
DGR	HIGH	0.28	0.23	−0.14
MZS	HIGH	0.29	0.23	−0.19
TAB	HIGH	0.31	0.25	−0.17
JCH	MID	0.29	0.23	−0.15
JSN	MID	0.28	0.23	−0.13
NWS	MID	0.31	0.23	−0.24
DMY	LOW	0.27	0.22	−0.14
CHJ	LOW	0.28	0.23	−0.13
ICH	LOW	0.29	0.23	−0.14

To quantify the contribution of the spatial and environmental matrices on the population differentiation, we conducted a multiple matrix regression with randomization (MMRR; Wang 2013) using *lgrMMRR()* in the *PopGenReport* package (Adamack and Gruber 2014). After MMRR, a commonality analysis (CA) was implemented using *commonalityCoeffieicient()* in the *yhat* package (Nimon et al. 2009), in order to figure out the contribution of multiple variables to patterns of genetic differentiation (Prunier et al. 2015), which was only for the significant spatial or environmental variables from the prior Mantel test. All figures in this study were visualized using the *ggplot2* package.

## 3 Results

### 3.1 Genetic Diversity and Population Differentiation

To characterize genetic diversity of *C. bursa‐pastoris* populations in the Korean Peninsula we genotyped 187 individuals from 10 populations at 233 RADseq loci. Genotype calling was performed using Stacks2‐PolyRAD, assuming bivalent inheritance for this allotetraploidy species. Genetic diversity across the populations was relatively high, despite the species being considered as predominant selfing plants. Observed heterozygosity (*H*_O_) ranged from 0.27 to 0.31, higher than expected heterozygosity (*H*_E_) which ranged from 0.22 to 0.25 (Table 1). Notably, all populations exhibited negative inbreeding coefficients (*F*_IS_ = −0.13 to −0.24), indicating an excess of heterozygotes relative to Hardy–Weinberg expectations. The negative *F*_IS_ values are inconsistent with high selfing, as selfing species typically have positive *F*_IS_ values. Instead, these negative values may reflect extensive outcrossing or heterozygote excess within populations.

**FIGURE 1:**
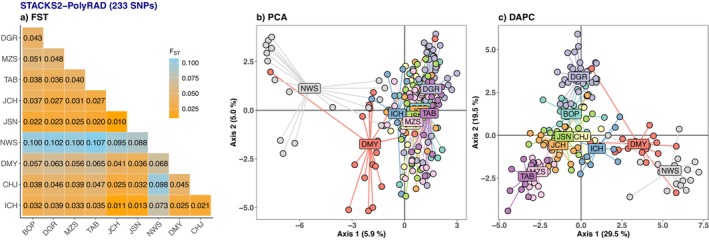
Population differentiation of *C. bursa‐pastoris* based on 233 genome‐wide SNPs. (a) Heatmap of pairwise genetic differentiation (FST) among the 10 populations. Blue colors indicate greater genetic differentiation. (b) Principal component analysis (PCA) and (c) Discriminant analysis of principal components (DAPC). Each point represents an individual, and colors indicate population identity. Population names are displayed in boxed labels positioned at the center of each population cluster.

Genetic differentiation between populations was generally low to moderate, with all populations having pairwise *F*_ST_ < 0.062, with the exception of NWS for which values ranged from 0.068 to 0.107, suggesting substantial gene flow among those populations, which the genetically low pairwise *F*_ST_ value (< 0.10) (Figure 1a). Their genetic differentiation patterns were also supported by ordination analyses using PCA and DAPC (Figure 1b,c). PCA showed that most individuals were genetically close to each other. Although the first two principal components explained only 10.9% of the total genetic variation, a subset of individuals from NWS and DMY was slightly separated from the remaining populations. DAPC, which combined PCA with discriminant analysis (DA) based on genetic variance between populations rather than the total variance of all individuals, also confirmed low gene differentiation among most populations. The first two discriminant axes of the DAPC together explained 49% of the genetic variation among populations, with only the NWS population showing partial separation, consistent with its relatively higher genetic differentiation.

We also performed analyses of molecular variance (AMOVA) to assess the distribution of genetic variation within and among the 10 populations and three altitudinal groups (Table S4). Consistent with the PCA and DAPC results, AMOVA indicated that most genetic variation occurred within populations, with only 12.7% of the total variance attributed to differences among populations and 87.3% occurring within populations (Table S4a). Similarly, altitudinal groupings had little influence on genetic variation: variation among populations within altitudinal groups accounts for 11.6% of the total variance, and variation within populations accounted for 87.6% (Table S4b). These results exhibited consistent weak genetic differentiation among all populations and altitudinal groups.

### 3.2 Genetic Structure of Populations

**FIGURE 2:**
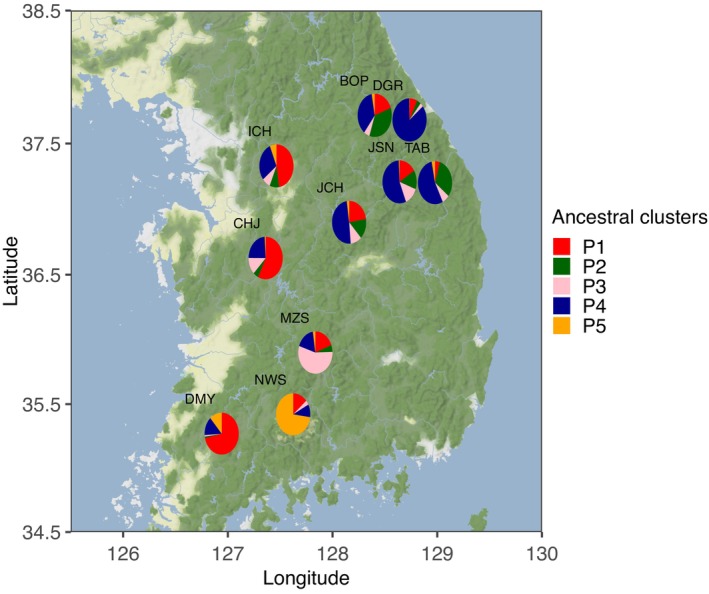
Mean ancestry composition of *C. bursa‐pastoris* populations under *K* = 5. Pie charts show average cluster proportions per population, based on 200 replicate runs. Colors indicate ancestral gene clusters.

Population clustering revealed that the 10 sampled populations form five ancestral genetic clusters (*K* = 5), as determined by the plateau in cross‐entropy criterion (Figure S2). To better understand how these gene clusters were distributed across populations, we also visualized the genetic admixtures under *K* = 3, 4, and 5 (Figure S3). To further clarify the genetic structure of admixed populations, we visualized the averaged composition of each ancestral gene cluster under *K* = 5 (Figure 2). Cluster 4 (green) was predominantly distributed in the northern regions; Cluster 1 (red) in the western populations (ICH, CHJ, and DMY); Cluster 2 (pink) in the east (BOP, TAB, and JSN); and Cluster 5 (blue) in the southern populations (DMY and NWS). Cluster 3 (orange) remains unclear, possibly due to the lack of sampling from the southeastern regions. All populations exhibited admixture across these genetically distinct ancestral clusters, indicating extensive gene flow across regions of the Korean Peninsula, potentially facilitated by anthropogenic dispersal.

### 3.3 Spatial and Environmental Drivers Contributing to Genetic Differentiation

**FIGURE 3:**
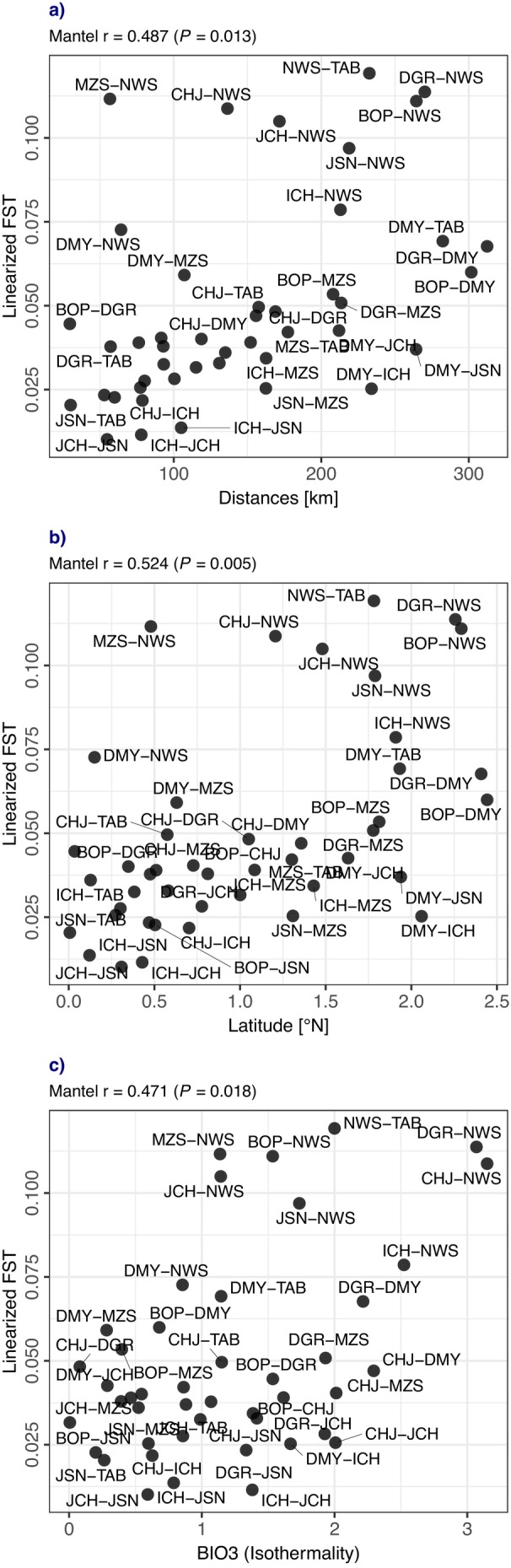
Genetic divergence associated with geographic and environmental factors. (a) Isolation by Distance (IBD), with pairwise genetic differentiation (*F*_ST_) plotted against geographic distance (km); (b) IBD against latitude; and (c) isolation by environment (IBE), with *F*_ST_ plotted against isothermality (BIO3). Partial Mantel test correlation coefficients are shown in each plot.

Mantel tests revealed significant correlations between population genetic differentiation and spatial/environmental variables, including latitude, geographic distance, and isothermality (BIO3) (Table 2, Table S3, and Figure 3). We also performed multiple matrix regression with randomization (MMRR) using two models that incorporated predictors that Mantel tests identified as associated with population differentiation: (i) geographic distance and isothermality, and (ii) latitude and isothermality. Both models were statistically significant. In the first model, both geographic distance and isothermality showed marginal effects (*β*_Distance_ = 2.89, *p* = 0.052; *β*_Isothermality_ = 2.71, *p* = 0.066), while in the second model, only latitude was significant (*β*_Latitude_ = 3.06, *p* = 0.032); isothermality was not (*β*_Isothermality_ = 2.42, *p* = 0.106). According to commonality analysis, each predictor accounted for > 60% of the explained genetic variation in both models (Table 3), suggesting that these predictors contribute substantially to the genetic differentiation of *C. bursa‐pastoris* in the Korean Peninsula. Notably, both predictors exhibited high unique and common contributions, implying collinearity between spatial and environmental predictors. Pairwise linear regression between spatial and environmental predictors revealed significant correlations between latitude and isothermality (*t* = 2.62, *p* = 0.012), and between geographic distance and isothermality (*t* = 2.13, *p* = 0.039).

**TABLE 2:** Results of Mantel tests (*n* = 9999 permutations) examining the relationships between genetic distance and spatial/environmental (BIO1–BIO19) distances. Mantel correlation coefficients (*r*) and *p* values are shown. Significant results (*p* < 0.05) are indicated in this table and the remaining variables listed in Table S3.

Spatial variables	Variables	*r*	*p*
	Latitudes	0.524	0.005
	Distances	0.487	0.013
Environmental variables	BIO3	0.471	0.018

**TABLE 3:** Summary of the multiple matrix regression with randomization (MMRR) combined with commonality analysis (CA), assessing the relative contributions of spatial and environmental distances to genetic differentiation (*F*_ST_) in *C. bursa‐pastoris* . Regression coefficients (*β*) and *p*‐values from the MMRR are also presented. (a) Geographic distance and isothermality (BIO3); (b) Latitudinal distance and isothermality (BIO3).

	Predictor	*β* (*t*)	Unique	Common	Total
(a) Model: *F*_*ST*_ ~ Geographical distance + BIO3					
*R* ^*2*^ = 0.35**	Distance	0.013 (2.89^†^)	0.129 (36.7%)	0.108 (32.5%)	0.234 (67.5%)
	BIO3	0.012 (2.71^†^)	0.114 (30.8%)	0.108 (32.5%)	0.222 (63.3%)
(b) Model: *F*_ST_ ~ Latitude + BIO3					
*R* ^*2*^ = 0.36**	Latitude	0.015 (3.06*)	0.142 (39.0%)	0.133 (36.7%)	0.275 (75.7%)
	BIO3	0.011 (2.42^ns^)	0.089 (24.3%)	0.133 (36.7%)	0.222 (61.0%)

*Note:* Significance levels: **p < 0.01, *p < 0.05, ^†^ *p* < 0.10, and ns, not significant (*p* ≥ 0.10).

Taken together, these results suggest that genetic differentiation among Korean *C. bursa‐pastoris* populations is associated with both spatial and climatic gradients. However, because spatial and climatic variables were strongly correlated, their independent effects could not be fully disentangled, indicating that both factors are closely associated with patterns of genetic differentiation.

## 4 Discussion

This study aimed to uncover how genetic diversity of *C. bursa‐pastoris* populations in Korea has been shaped by the combined impacts of anthropogenic activities and environmental gradients. We found that *C. bursa‐pastoris* populations in the Korean Peninsula exhibit relatively low genetic differentiation among populations and high genetic diversity within populations. Despite geographical separation and ecological variation, populations in the Korean Peninsula have experienced extensive gene flow. We propose that human‐mediated dispersal may play a major role in shaping the population structure of *C. bursa‐pastoris in Korea*.

### 4.1 Unexpectedly High Possibility of Outcrossing in Korean *C. bursa‐pastoris* Populations

*Capsella bursa‐pastoris* is well known as a predominantly selfing species (Hintz et al. 2006; Hurka and Neuffer 1997), however, our study revealed high heterozygosity and negative inbreeding coefficients (Table 1), suggesting the possibility of outcrossing within and high gene flow among populations. Interestingly, similar patterns of negative inbreeding coefficients have been observed in other predominantly selfing or self‐compatible species (Brandrud et al. 2017; Carleial et al. 2017; Cordeiro et al. 2019; Toker et al. 2025). For example, Brandrud et al. (2017) analyzed the tetraploid *Cochearia officinalis* (Brassicaceae) using RADseq and found negative *F*_IS_ values across all tested populations. They suggested that variation in *F*_IS_ among populations reflected differences in mating systems or levels of gene flow. Specifically, coastal and shoreline populations exhibited more negative *F*_IS_ values, likely due to increased gene flow, whereas inland populations had *F*_IS_ values closer to zero. In contrast, our study revealed negative *F*_IS_ values across all populations, with little differences among them. This similar level implies that all populations may experience consistently high levels of gene flow. Furthermore, such negative inbreeding coefficients could result from disassortative mating, selection favoring heterozygosity, or the purging of homozygosity through inbreeding depression (Charlesworth and Charlesworth 1987).

In contrast to previous studies of European populations, which reported low observed heterozygosity and high inbreeding coefficient (often excessing 0.9) consistent with predominant selfing (Wesse et al. 2021), Korean population exhibited comparatively high heterozygosity and negative *F*_IS_ values. These results suggest that the mating system and population genetic structure of Korean populations may differ substantially from those reported across much of the species' introduced regions. Such differences may reflect regional variation in demographic history, gene flow, or ecological conditions within this study.

Previous studies also observed the possibility of outcrossing in *C. bursa‐pastoris* . Outcrossing rates across European populations from Scandinavia, Central Europe, the British, Mediterranean countries, and the Middle East, have been reported to range from 0% to 20% under greenhouse conditions (Hurka et al. 1989; Hurka and Neuffer 1997). During the colonization of *C. bursa‐pastoris* populations within Eurasia, variability in selfing and outcrossing has been postulated (Cornille et al. 2016). Furthermore, a global‐scale study by Wesse et al. (2021), which sampled *C. bursa‐pastoris* populations across North and South America, northern and southern Africa, Australia, Europe, Central Asia, and parts of East Asia, revealed low but consistent outcrossing rates. Taken together, our results, along with previous large‐scale studies of *C. bursa‐pastoris* , suggest this species may exhibit considerable outcrossing in the Korean populations.

### 4.2 Anthropogenic and Ecological Factors Facilitating Outcrossing

In self‐compatible plants, population density may affect the selfing rate (Ågren and Sletvold 2025), influencing the mating variability by providing opportunities for outcrossing (Karron et al. 1995). Indeed, in Korea *C. bursa‐pastoris* populations, most individuals within each sampled population were observed to grow at high density because of high demand based on Korean culture, with inflorescences often overlapping and in close contact at late flowering season (B. Choi, field observations). Physical close distance between individuals in such densely populated micro‐habitat may strongly promote pollen exchanges among neighboring individuals, which can increase the chances of outcrossing.

In addition, flowering time and inflorescence development can also influence outcrossing by promoting residual outcrossing. For example, *Medicago truncatula* , a predominantly selfing species, maintains a low but significant level of residual outcrossing (Jullien et al. 2021). This pattern is largely shaped by temporal variation in flower development, in which early season flowers tend to remain in a closed state for a longer period through forming pods earlier, limiting outcrossing opportunities, whereas later season flowers open more exposed to external pollen sources. In our study, we sampled individuals with spatial intervals within each population to maintain independent maternal genotypes. However, the sampling was conducted near the end of the flowering season, when flowers are likely to be exposed to neighboring external pollen, suggesting that individuals had already experienced residual outcrossing in the field prior to seed collection. Taken together, the observed outcrossing may reflect seasonally mediated ecological opportunities for residual outcrossing, rather than a shift in the mating system of Korean *C. bursa‐pastoris* populations.

Lastly, however, we could not exclude the possibility that heterozygosity may be influenced by the allotetraploid genomic structure of *C. bursa‐pastoris* . Although the subgenomes are overall quite similar, they exhibit approximately ~3% sequence divergence (Kryvokhyzha et al. 2019). In this study, we conducted *de novo* assembly without separating subgenomes as well as alignment to reference genomes, following the approach of Kryvokhyzha et al. (2019). Therefore, the heterozygosity may have been overestimated, potentially leading to negative *F*_IS_ values even in the absence of frequent outcrossing, which seems like analytical artifacts related to allotetraploidy.

Nevertheless, the consistent ecological interpretations derived from the genetic diversity patterns across Korean *C. bursa‐pastoris* populations in this study suggest that multiple factors have played a significant role in shaping their mating strategies. The patterns of high heterozygosity in this predominantly selfing species may be interpreted as evidence of potential outcrossing, driven by human activity and other ecological patterns happening in the Korean Peninsula. Taken together, such outcrossing may help mitigate long‐term evolutionary constraints such as the accumulation of deleterious mutations and inbreeding depression (Winn et al. 2011; Wright et al. 2013), by providing an alternative evolutionary strategy for predominantly selfing species within fine‐scale regional habitats.

### 4.3 Low Genetic Differentiation Among Populations

Genetic clustering results (Figures 1 and 2, and Figure S3) showed low genetic differentiation and admixture among five ancestral clusters across 10 populations, suggesting historical colonization of the Korean Peninsula through extensive gene flow based on high demand by Korean people, further implying that genetic exchange between populations has occurred. These patterns of diversity also contradict the typical patterns of predominant selfing plants.

Based on Approximate Bayesian Computation (ABC) analysis, Cornille et al. (2016) suggested that *C. bursa‐pastoris* recently and rapidly colonized Asia from the Middle East approximately 1000 years ago, eventually reaching the far eastern edge of the continent, including the Korean Peninsula. Although our study cannot trace how the current genetic clusters formed after this initial introduction, two potential scenarios can be assumed: (1) multiple introductions of genetically distinct genotypes across different regions, or (2) a limited number of founding genotypes introduced locally, followed by local divergence. In either case, subsequent extensive gene flow likely shaped by Korean cultural bases appears to have admixture genetic structure, resulting in low genetic differentiation. Interestingly, at least two genetic clusters originating from Europe and Asia were reported to have colonized in the northern region of the Korean Peninsula (Cornille et al. 2016). Their findings suggest that at least two distinct genetic clusters from Europe and Asia co‐existed nearby the peninsula, and consequently also may have been introduced multiple times into the Korean Peninsula at that close time.

Taken together, these historical and cultural factors provide a reasonable explanation for the relatively low to moderate levels of genetic differentiation observed in this study where the highest pairwise *F*_ST_ value did not exceed 0.107 (Figure 1a) and AMOVA results (Table S4) as well as the overall admixture pattern across populations (Figure 2 and Figure S3). These spatially structured patterns suggest that regional genetic components may have been established prior to genetic differentiation within the Korean Peninsula and further support the hypothesis of multiple introductions followed by intra‐peninsular gene flow by anthropogenic activities. Thus, the genetic structures of *C. bursa‐pastoris* in the Korean Peninsula can be understood within these broader historical, geopolitical, and cultural contexts, shaped by human activities across both past and modern times.

### 4.4 Influences of Spatial and Environmental Predictors on the Differentiation

Although Korean *C. bursa‐pastoris* populations exhibit relatively low levels of genetic differentiation, our results suggest that both spatial and environmental variables play a significant role in shaping their genetic divergence (Tables 2 and 3). Previous research has reported a significant positive correlation between linearized *F*_ST_ and geographic distance across the Eurasia continent (*r* = 0.31, *p* < 0.001), especially between three main genetic clusters (EUR, ASI, and ME) (Cornille et al. 2016). However, within each region, no significant correlation was observed, suggesting that spatial effects on genetic differentiation are more pronounced between broad genetic clusters than between local populations within those regions. Similarly, Wesse et al. (2021) found significant correlations between genetic and geographic distances within Europe, but all models, except for Scandinavia, showed very low explanatory power (*R* ^2^ < 0.06), reinforcing the notion of limited spatial structure within regions. In the European‐Mediterranean region, 52 accessions showed stronger and highly significant correlations in isolation‐by‐distance effects (*r* = 0.49, *p* < 0.001) (Ceplitis et al. 2005).

Given these previous patterns, we initially expected limited spatial impact on Korean *C. bursa‐pastoris* populations. Surprisingly, however, we found that spatial effects on genetic variation were significant. Notably, altitudinal groups or elevation gradients did not significantly explain genetic divergence, suggesting that strong gene flow across the Korean Peninsula may override fine‐scale altitudinal gradient structure rather than geographical distances.

On the other hand, in the same large scale study (Wesse et al. 2021), genetic clusters of *C. bursa‐pastoris* have been associated with distinct macroclimatic variables such as BIO3 (isothermality), BIO6 (minimum temperature of coldest month), and BIO18 (precipitation of warmest quarter). Among these, isothermality was one of the most discriminating climatic factors and was the only variable that significantly explained genetic variation in our study. To further understand how isothermality contributes to genetic variation, future studies could implement genome‐wide association studies (GWAS) or QTLs focusing on germination and flowering traits across populations. Such approaches would help clarify how genetic variation drives phenotypic traits under environmental selection related to temperature.

Taken all together, we propose that populations of *C. bursa‐pastoris* across the Korean Peninsula exhibit low levels of genetic differentiation, likely shaped by anthropogenic activities that have facilitated gene flow among populations, coupled with presumable gene exchanges through outcrossing. Furthermore, despite the relatively narrow and local geographic scale, genetic variation among populations appears to be significantly influenced by both spatial and climatic factors. However, whether these patterns reflect signals of local adaptation or simply population structure shaped by environmental gradients remains unclear, highlighting the need for further studies to determine adaptive divergence and ecological responses of Korean *C. bursa‐pastoris* in comparison to neighboring populations from other East Asia countries.

## Author Contributions

**Byungwook Choi:** conceptualization (equal), formal analysis (lead), funding acquisition (equal), methodology (lead), visualization (lead), writing – original draft (lead), writing – review and editing (equal). **Eunsuk Kim:** conceptualization (equal), funding acquisition (equal), supervision (equal), writing – review and editing (equal). **Peter Tiffin:** funding acquisition (equal), supervision (equal), writing – review and editing (equal). **Sang‐Tae Kim:** conceptualization (equal), supervision (equal), writing – review and editing (equal).

## Funding

This work was supported by the National Science Foundation (IOS‐2243817), National Research Foundation of Korea (RS‐2020‐NR050221 and RS‐2025‐00558787), and the GIST Research Project grant funded by the GIST in 2026.

## Conflicts of Interest

The authors declare no conflicts of interest.

## Supporting information


**Data S1:** ece373980‐sup‐0001‐supinfo.docx.

**Figure S1:** Map representing the natural populations of *Capsella bursa‐pastoris* in the Korean peninsula. The information of each population marked in Table S1 according to their corresponding population ID.
**Figure S2:** Cross‐entropy values for K (number of gene clusters) ranging from *K* = 1–20. The optimal number was determined as the point where the cross‐entropy values began to plateau.
**Figure S3:** Population structure of Capsella bursa‐pastoris in the Korean Peninsula, inferred using the snmf() from LEA R package. (top) Individual assignment plots for each population based on *K* = 3–5 ancestral clusters. The ancestral clusters are reported at the individual level, and populations are labeled according to the IDs defined in Table S1.

**Table S1:** Geographical information for the 10 natural populations of *C. bursa‐pastoris* analyzed in this study. Latitude, longitude, elevation (in meters above sea level), altitudinal group, and number of individuals sequenced (*N*) are provided.
**Table S2:** Information of 19 environmental variables defined by the WorldClime database.
**Table S3:** Results of Mantel tests (*n* = 9999 permutations) examining the relationships between genetic distance and spatial/environmental (BIO1–BIO19) distances. Mantel correlation coefficients (*r*) and *p* values are shown. Significant results (*p* < 0.05) are indicated in bold.
**Table S4:** Results of analysis of molecular variance (AMOVA) for C. bursa‐pastoris: (a) genetic differentiation among populations, and (b) genetic differentiation among altitudinal groups, with populations nested within groups.

**Dataset S1:** Mean read counts and coverage of individuals across all tested populations.

## Data Availability

RADseq assemblies can be accessed at NCBI BioProject PRJNA1423438 (https://www.ncbi.nlm.nih.gov/sra/PRJNA1423438). R scripts for this study are available on FigShare (https://doi.org/10.6084/m9.figshare.32737797).
