# Supplementary material for: Local Genetic Signatures of a Global Weed: Population Structure and Ecological Drivers of Capsella bursa‐pastoris Diversity in the Korean Peninsula
Source: Ecol Evol. 2026 Aug 18;16(8):e73980. doi: 10.1002/ece3.73980 (PMC13487376; doi:10.1002/ece3.73980)
Supplement: Supplementary file 1 [file ECE3-16-e73980-s001.docx]

**Supplemental Information**

**Local Genetic Signatures of a Global Weed: Population Structure and Ecological Drivers of *Capsella bursa-pastoris* diversity in the Korean Peninsula**

**Authors:** Byungwook Choi^1,2^**^*^**, Eunsuk Kim^2^, Peter Tiffin^1^, and Sang-Tae Kim^3^**^*^**

Byungwook Choi (choib@umn.edu) (https://orcid.org/0000-0002-6134-9172)

Eunsuk Kim (eunsukkim@gist.ac.kr) (https://orcid.org/0000-0001-8059-7334)

Peter Tiffin (ptiffin@umn.edu) (https://orcid.org/0000-0003-1975-610X)

Sang-Tae Kim (stkim@catholic.ac.kr) (https://orcid.org/0000-0003-1645-6021)

**Affiliations:** ^1^Department of Plant and Microbial Biology, University of Minnesota, St. Paul, MN, USA; ^2^Department of Environmental and Energy Engineering, Gwangju Institute of Science and Technology, Gwangju, Republic of Korea; ^3^Department of Medical and Biological Sciences, The Catholic University of Korea, Bucheon, Republic of Korea

***Corresponding authors**

Byungwook Choi (choib@umn.edu)

Sang-Tae Kim (stkim@catholic.ac.kr)

***Library Preparation and Sequencing***

Briefly, 50 ~ 60 mg of fresh leaves of *C. bursa-pastoris* were put in a 1.5 Eppendorf tube, followed by freeze with liquid nitrogen for 30 seconds, and were homogenized using Tissue Lyser II (Qiagen, Hilden, Germany) with 20/s speed for five times repeats after adding sterile beads to the tube. For cell lysis, 500 μl of high salt DNA extraction buffer (200 mM Tris-HCl, 1.5 M NaCl, 25 mM EDTA, 0.5 % SDS, and 0.16 % 2-Mercaptoethanol) was added to the homogenized mixture, and mixed with vortex for 30 seconds. The mixture was centrifuged at 13,000 rpm for 60 seconds, and then the supernatant was transferred to a new tube. The supernatant was mixed with an equal volume of chloroform:Isoamyl alcohol (24:1) (#C0549, Sigma-Aldrich) and vortexed for 30 seconds and centrifuged at 13,000 rpm for 60 seconds. The clear aqueous supernatant was transferred to a new tube, an equal volume of isopropanol was added and mixed, and left at room temperature for five minutes. After centrifugation of the mixture at 13,000 rpm for 60 seconds, the supernatant was carefully taken off and discarded and the pellet was dried for 30 minutes. Finally, the pellet was eluted with a 30 μl of TE buffer. Quantification and quality of DNA were measured using Eppendorf Biospectrometer (Eppendorf, Hamburg, Germany), and confirm its size and degradation on gel electrophoresis. Individuals with unfragmented DNA higher than 10 k bases were used for library preparation.

200 ng of DNA was digested in a 30 μl volume using 1 μl of KpnI-HF (#R3142, NEB) restriction enzyme and 3 μl of the 10x reaction buffer, and incubate in a thermal cycler at 37 ℃ for 30 minutes. The digested DNA was performed ligation with 1 μl of 0.05 μM KpnI adapters (barcodes) in 30 μl volume with 3 μl of 50 % PEG 4000, 3 μl of the 10 x reaction buffer, 12 μl of nuclease-free water, and 1 μl (5U) of T4 ligase. Ligation was reacted at room temperature for 30 minutes, followed by a 65 ℃ in a thermal cycler for 20 minutes to inactivate the enzyme reaction. Each 15 μl of 187 barcoded samples was combined in a sterile 2 ml collection tube and mixed by pipetting well. The 187-plex pooled sample was purified with SPRI beads, and eluted to 30 μl of nuclease-free water to concentrate DNA. Pooled DNA was fragmented using a sonicator (Q800R2, Qsonica, USA) with conditions of 20 % of amplitude and 15 / 15 seconds of pulse on / off for 5 minutes. Sheared DNA was end-repaired in 100 μl of volume with 5 μl of End Repair Enzyme mix (#E6050, NEB), 10 μl of 10x reaction buffer, 25 ul of sheared DNA and 60 ul of nuclease-free water in a thermal cycler at 20 ℃ for 30 minutes. After SPRI beads purification, end-repaired DNA was dA-tailed in 50 μl of volume with 3 μl of Klewnow Fragment, 5 μl of reaction buffer, 30 μl of end-repaired DNA, and 12 ul of nuclease water in a thermal cycler at 37 ℃ for 30 minutes. After SPRI beads purification, dA-tailed DNA was ligated with universal adapter set using Quick T4 Ligase (#E6056, NEB) in a thermal cycler at 20 ℃ for 15 minutes. The universal adapters provided complementary sequences to bind universal primers for PCR. DNA amplification was performed with 1 μl of the forward and reverse PCR primer (10 μM), 5 μl of universal-adapter ligated DNA, 15 μl of 2x Phusion HF Mater Mix (#M0541, NEB), and 8 μl of nuclease-free water in a thermal cycler (TP600, Takara) with conditions of 98 ℃ for 30 seconds, 14 cycles of 98 ℃ for 10 seconds, 65 ℃ for 30 seconds, 72 ℃ for 30 seconds, and 72 ℃ for 5 min, and hold at 10 ℃. After final SPRI-beads purification, prepared library was quantified and checked amplicon size (Mean size; 543.75 bp) using DNA 1000 kit (#5067-1504) for Agilent Bioanalyzer 2100 (Agilent Technologies, CA, USA). Illumina sequencing was performed using Hiseq2500 50SE by Macrogen Inc. (Korea).
