# Supplementary material for: Local Genetic Signatures of a Global Weed: Population Structure and Ecological Drivers of Capsella bursa‐pastoris Diversity in the Korean Peninsula
Source: Ecol Evol. 2026 Aug 18;16(8):e73980. doi: 10.1002/ece3.73980 (PMC13487376; doi:10.1002/ece3.73980)
Supplement: Supplementary file 2 [file ECE3-16-e73980-s003.docx]

**Supplemental Tables**

**Local Genetic Signatures of a Global Weed: Population Structure and Ecological Drivers of *Capsella bursa-pastoris* diversity in the Korean Peninsula**

**Authors:** Byungwook Choi^1,2^**^*^**, Eunsuk Kim^2^, Peter Tiffin^1^, and Sang-Tae Kim^3^**^*^**

Byungwook Choi (choib@umn.edu) (https://orcid.org/0000-0002-6134-9172)

Eunsuk Kim (eunsukkim@gist.ac.kr) (https://orcid.org/0000-0001-8059-7334)

Peter Tiffin (ptiffin@umn.edu) (https://orcid.org/0000-0003-1975-610X)

Sang-Tae Kim (stkim@catholic.ac.kr) (https://orcid.org/0000-0003-1645-6021)

**Affiliations:** ^1^Department of Plant and Microbial Biology, University of Minnesota, St. Paul, MN, USA; ^2^Department of Environmental and Energy Engineering, Gwangju Institute of Science and Technology, Gwangju, Republic of Korea; ^3^Department of Medical and Biological Sciences, The Catholic University of Korea, Bucheon, Republic of Korea

***Corresponding authors**

Byungwook Choi (choib@umn.edu)

Sang-Tae Kim (stkim@catholic.ac.kr)

**List of Supplemental Tables**

**Table S1**. Geographical information for the ten natural populations of *C. bursa-pastoris* analysed in this study. Latitude, longitude, elevation (in meters above sea level), altitudinal group, and number of individuals sequenced (N) are provided.

**Table S2.** Information of nineteen environmental variables defined by the WorldClime database.

**Table S3.** Results of Mantel tests (n = 9,999 permutations) examining the relationships between genetic distance and spatial/environmental (BIO1–BIO19) distances. Mantel correlation coefficients (*r*) and *P values* are shown. Significant results (*p* < 0.05) are indicated in bold.

**Table S4.** Results of analysis of molecular variance (AMOVA) for *C. bursa-pastoris*: (a) genetic differentiation among populations, and (b) genetic differentiation among altitudinal groups, with populations nested within groups.

**Table S1**. Geographical information for the ten natural populations of *C. bursa-pastoris* analysed in this study. Latitude, longitude, elevation (in meters above sea level), altitudinal group, and number of individuals sequenced (N) are provided.

| ID | Populations | Latitude | Longitude | Groups | Elevation | N |
| --- | --- | --- | --- | --- | --- | --- |
| BOP | Bongpyeong | 37.42'45.7"N | 128.24'10.0"E | High | 719 | 20 |
| DGR | Daegwallyeong | 37.40'40.7"N | 128.44'06.5"E | High | 755 | 36 |
| MZS | Muju | 35.54'00.8"N | 127.50'10.3"E | High | 862 | 24 |
| TAB | Taebaek | 37.12'08.8"N | 128.58'44.3"E | High | 775 | 15 |
| JCH | Jecheon | 36.54'03.9"N | 128.09'31.0"E | Mid | 326 | 16 |
| JSN | Jeongseon | 37.12'31.5"N | 128.38'24.1"E | Mid | 386 | 13 |
| NWS | Namwon | 35.25'14.9"N | 127.37'27.5"E | Mid | 345 | 19 |
| DMY | Damyang | 35.16'12.5"N | 126.56'29.0"E | Low | 30 | 19 |
| CHJ | Cheongju | 36.37'38.8"N | 127.21'41.0"E | Low | 57 | 13 |
| ICH | Icheon | 37.19'48.0"N | 127.27'51.3"E | Low | 68 | 12 |

**Table S2.** Information of nineteen environmental variables defined by the WorldClime database.

| Bioclimatic variables | Description |
| --- | --- |
| BIO1 | Annual Mean Temperature |
| BIO2 | Mean Diurnal Range (Mean of monthly Temp_Max_ - Temp_Min_) |
| BIO3 | Isothermality (BIO2/BIO7 * 100) |
| BIO4 | Temperature Seasonality (Standard deviation * 100) |
| BIO5 | Max Temperature of Warmest Month |
| BIO6 | Min Temperature of Coldest Month |
| BIO7 | Temperature Annual Range (BIO5 - BIO6) |
| BIO8 | Mean Temperature of Wettest Quarter |
| BIO9 | Mean Temperature of Driest Quarter |
| BIO10 | Mean Temperature of Warmest Quarter |
| BIO11 | Mean Temperature of Coldest Quarter |
| BIO12 | Annual Precipitation |
| BIO13 | Precipitation of Wettest Month |
| BIO14 | Precipitation of Driest Month |
| BIO15 | Precipitation Seasonality (Coefficient of Variation) |
| BIO16 | Precipitation of Wettest Quarter |
| BIO17 | Precipitation of Driest Quarter |
| BIO18 | Precipitation of Warmest Quarter |
| BIO19 | Precipitation of Coldest Quarter |

**Table S3.** Results of Mantel tests (n = 9,999 permutations) examining the relationships between genetic distance and spatial/environmental (BIO1–BIO19) distances. Mantel correlation coefficients (*r*) and *P values* are shown. Significant results (*p* < 0.05) are indicated in bold.

| Spatial variables | Variables | r | *P* value |
| --- | --- | --- | --- |
|  | Altitudes | 0.038 | 0.406 |
|  | Latitudes | **0.524** | **0.005** |
|  | Longitudes | 0.135 | 0.223 |
|  | Distances | **0.487** | **0.013** |
| Environmental variables | BIO1 | -0.008 | 0.468 |
|  | BIO2 | 0.147 | 0.212 |
|  | BIO3 | **0.471** | **0.018** |
|  | BIO4 | -0.222 | 0.894 |
|  | BIO5 | 0.091 | 0.203 |
|  | BIO6 | -0.158 | 0.694 |
|  | BIO7 | -0.141 | 0.691 |
|  | BIO8 | 0.050 | 0.333 |
|  | BIO9 | -0.073 | 0.540 |
|  | BIO10 | 0.015 | 0.403 |
|  | BIO11 | -0.073 | 0.536 |
|  | BIO12 | -0.086 | 0.605 |
|  | BIO13 | -0.105 | 0.633 |
|  | BIO14 | -0.245 | 0.941 |
|  | BIO15 | -0.172 | 0.841 |
|  | BIO16 | -0.105 | 0.622 |
|  | BIO17 | -0.245 | 0.938 |
|  | BIO18 | 0.001 | 0.452 |
|  | BIO19 | -0.245 | 0.935 |

**Table S4.** Results of analysis of molecular variance (AMOVA) for *C. bursa-pastoris*: (a) genetic differentiation among populations, and (b) genetic differentiation among altitudinal groups, with populations nested within groups.

| (a) Populations | Df | SumSq | MeanSq | Variance | % |
| --- | --- | --- | --- | --- | --- |
| Between populations | 9 | 636.69 | 70.74 | 2.83 | 12.21 |
| Within populations | 171 | 3478.92 | 20.34 | 20.34 | 87.79 |
| Total | 180 | 4115.61 | 22.86 | 23.17 | 100.00 |

| (b) Altitudinal groups | Df | SumSq | MeanSq | Variance | % |
| --- | --- | --- | --- | --- | --- |
| Between altitudes | 2 | 157.90 | 78.95 | 0.21 | 0.90 |
| Between samples within altitudes | 7 | 478.79 | 68.40 | 2.68 | 11.55 |
| Within samples | 171 | 3478.92 | 20.34 | 20.35 | 87.55 |
| Total | 180 | 4115.61 | 22.86 | 23.24 | 100.00 |
