# Supplementary material for: Local Genetic Signatures of a Global Weed: Population Structure and Ecological Drivers of Capsella bursa‐pastoris Diversity in the Korean Peninsula
Source: Ecol Evol. 2026 Aug 18;16(8):e73980. doi: 10.1002/ece3.73980 (PMC13487376; doi:10.1002/ece3.73980)
Supplement: Supplementary file 3 [file ECE3-16-e73980-s004.docx]

**Supplemental Figures**

**Local Genetic Signatures of a Global Weed: Population Structure and Ecological Drivers of *Capsella bursa-pastoris* diversity in the Korean Peninsula**

**Authors:** Byungwook Choi^1,2^**^*^**, Eunsuk Kim^2^, Peter Tiffin^1^, and Sang-Tae Kim^3^**^*^**

Byungwook Choi (choib@umn.edu) (https://orcid.org/0000-0002-6134-9172)

Eunsuk Kim (eunsukkim@gist.ac.kr) (https://orcid.org/0000-0001-8059-7334)

Peter Tiffin (ptiffin@umn.edu) (https://orcid.org/0000-0003-1975-610X)

Sang-Tae Kim (stkim@catholic.ac.kr) (https://orcid.org/0000-0003-1645-6021)

**Affiliations:** ^1^Department of Plant and Microbial Biology, University of Minnesota, St. Paul, MN, USA; ^2^Department of Environmental and Energy Engineering, Gwangju Institute of Science and Technology, Gwangju, Republic of Korea; ^3^Department of Medical and Biological Sciences, The Catholic University of Korea, Bucheon, Republic of Korea

***Corresponding authors**

Byungwook Choi (choib@umn.edu)

Sang-Tae Kim (stkim@catholic.ac.kr)

**List of Supplemental Figures**

**Figure S1.** Map representing the natural populations of *C. bursa-pastoris* in the Korean peninsula. The information of each population marked in **Table S1** according to their corresponding population ID.

**Figure S2**. Cross-entropy values for *K* (number of gene clusters) ranging from *K* = 1 to 20. The optimal number was determined as the point where the cross-entropy values began to plateau.

**Figure S3.** Population structure of *C. bursa-pastoris* in the Korean Peninsula, inferred using the *snmf*() from LEA R package. (top) Individual assignment plots for each population based on *K*=3 to 5 ancestral clusters. The ancestral clusters are reported at the individual level, and populations are labeled according to the IDs defined in **Table S1**.

**Figure S1.** Map representing the natural populations of *C. bursa-pastoris* in the Korean peninsula. The information of each population marked in **Table S1** according to their corresponding population ID.

**Figure S2**. Cross-entropy values for *K* (number of gene clusters) ranging from *K* = 1 to 20. The optimal number was determined as the point where the cross-entropy values began to plateau.

**Figure S3.** Population structure of *C. bursa-pastoris* in the Korean Peninsula, inferred using the *snmf*() from LEA R package. (top) Individual assignment plots for each population based on *K*=3 to 5 ancestral clusters. The ancestral clusters are reported at the individual level, and populations are labeled according to the IDs defined in **Table S1**.
